# Wave Vector Difference of Magnetic Bragg Reflections and Low Energy Magnetic Excitations in Charge-stripe Ordered La_2_NiO_4.11_

**DOI:** 10.1038/s41598-019-50904-8

**Published:** 2019-10-08

**Authors:** P. G. Freeman, S. R. Giblin, M. Skoulatos, R. A. Mole, D. Prabhakaran

**Affiliations:** 1 Jeremiah Horrocks Institute for Mathematics, Physics, and Astronomy, University of Central Lancashire, Preston, PR1 2HE UK; 2 Laboratory for Quantum Magnetism, Ecole Polytechnique Fédérale de Lausanne (EPFL), CH-1015 Lausanne, Switzerland; 3 Cardiff School of Physics and Astronomy, Queens Building, The Parade, Cardiff University, Cardiff, CF24 3AA UK; 4 Laboratory for Neutron Scattering, Paul Scherrer Institute, Villigen PSI, Villigen, Switzerland; 5 Physik Department E21, Technische Universität München, James-Franck-Str., 85747 Garching, Germany; 6 Heinz Maier-Leibnitz Zentrum, Lichtenbergstraße, 1, 85747 Garching, Germany; 7 Australian Nuclear Science and Technology Organisation, New Illawarra Rd, Lucas Heights, NSW Australia; 8 Department of Physics, Clarendon Laboratory, Oxford University, Oxford, OX1 3PU UK

**Keywords:** Magnetic properties and materials, Superconducting properties and materials

## Abstract

We report on the magnetism of charge-stripe ordered La_2_NiO_4.11±0.01_ by neutron scattering and *μ*SR. On going towards zero energy transfer there is an observed wave vector offset in the centring of the magnetic excitations and magnetic Bragg reflections, meaning the excitations cannot be described as Goldstone modes of the magnetic order. Weak transverse field μSR measurements determine the magnetically order volume fraction is 87% from the two stripe twins, and the temperature evolution of the magnetic excitations is consistent with the low energy excitations coming from the magnetically ordered volume of the material. We will discuss how these results contrast with the proposed origin of a similar wave vector offset recently observed in a La-based cuprate, and possible origins of this effect in La_2_NiO_4.11_.

## Introduction

When charge-stripe order was discovered in a non-superconducting La-based cuprate, it was compared to the charge-stripe order of insulating non-superconducting La_2−*x*_Sr_*x*_NiO_4+*δ*_ (LSNO)^1^. Since this discovery, the La-based cuprates have often been compared and contrasted to issostructural charge ordered materials in order to gain insights for our understanding of high temperature cuprate superconductivity. The striking discovery of the near universal hourglass shaped magnetic excitation spectrum of the hole doped cuprates however brought into question the relevance of understanding charge-stripe ordered LSNO, whose magnetic excitation spectrum are the typical spin wave cone shape of an antiferromagnet^2–4^. That was until the recent discovery of a highly similar hourglass shaped magnetic excitation spectrum in the insulating non-superconducting charge ordered cobaltite material La_2−*x*_Sr_*x*_CoO_4+*δ*_ (LSCoO)^5^. As LSNO has a single charge-stripe ordered phase below half doping, it is a simpler material to study than La_2−*x*_Sr_*x*_CoO_4+*δ*_ (LSCoO) which appears to phase separate into different charge ordered phases^6–9^.

Recently, a striking observation was reported on the magnetism in La_2_CuO_4+*δ*_ (LCO+O). On going towards zero energy transfer the magnetic excitations were observed to have a different wave vector centring compared to the magnetic Bragg reflections^10^, an offset. The magnetic excitations are not acting as Goldstone modes of a symmetry breaking magnetic order, which would have the same wave vector centring at lowest energy, that they were thought to be. As La-based cuprates are known not to fully magnetically order^11,12^, the offset of the magnetic excitations in LCO+O was attributed to the lowest energy magnetic excitations occurring in unordered magnetic volume of the material, instead of the magnetically ordered volume of the material^10^. In this report we will show there is a similar offset in wave vector between the lowest energy magnetic excitations and the magnetic Bragg reflections in tetragonal charge-stripe ordered La_2_NiO_4.11_, and that these magnetic excitations have to occur in the magnetically ordered phase of the material^13,14^.

**Figure 1:**
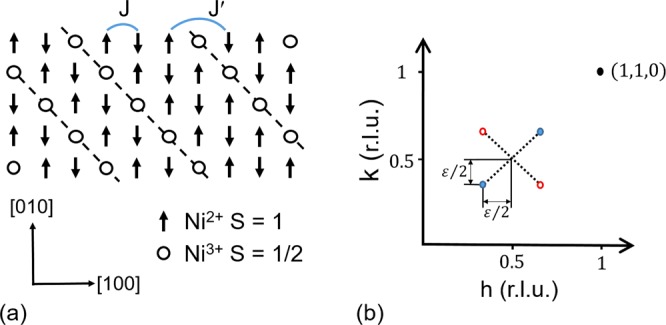
Charge-stripe order of La_2−*x*_Sr_*x*_NiO_4+*δ*_. (**a**) A model of Ni-centred charge-stripe order in a single Ni-O layer of La_2−*x*_Sr_*x*_NiO_4+*δ*_ (LSNO), where the average periodicity 1/$\varepsilon$ is achieved by varying the spacing between the charge-stripes. In this figure the O sites are omitted for the purpose of clarity. We indicate the intra-stripe *J*, and inter-stripe spin interactions *J*′ that are required to model the spin wave excitation spectrum of La_2−*x*_Sr_*x*_NiO_4+*δ*_^4,14^. (**b**) The position of magnetic Bragg reflections in the (*HK*0) plane of reciprocal space for the magnetism of charge-striped ordered La_2−*x*_Sr_*x*_NiO_4+*δ*_. The charge-stripe order shown in (**a**) produces the magnetic Bragg reflections identified by the solid blue symbols in (**b**). For tetragonal La_2−*x*_Sr_*x*_NiO_4+*δ*_ there is a second charge-stripe domain with charge-stripes rotated by 90 degrees in comparison to (**a**), and this twin produces the magnetic Bragg reflections identified by open red symbols in (**b**).

In LSNO holes are introduced into the Ni-O layers either by substitution of La^3+^ by Sr^2+^, or by the addition of excess oxygen, $\delta  > 0$, to achieve a hole doping level of ${n}_{h}=x+2\delta$. On cooling, these holes order into lines of charges in the Ni-O layers, charge-stripes, that are oriented at 45° to the Ni-O bonds with the charge-stripes aligned to be parallel in adjacent Ni-O layers^15,16^. At a lower temperature, the Ni^2+^, $S=1$ spins order antiferromagnetically between the charge-stripes which act as antiphase domain walls^15^. The Ni^2+^ ordered spin sites have two electrons in the ${e}_{g}$ orbital. A Ni centred charge-stripe (site centred) is a Ni^3+^ sites that has a lone ${e}_{g}$ electron that is frustrated from ordering within the Ni-O plane by charge-stripes being anitphase domain walls. In Fig. 1(a) we show a Ni centred charge-stripe order model of a single Ni-O layer of LSNO, which produces magnetic Bragg reflections at $(h+(1\pm \varepsilon )/2,k+(1\pm \varepsilon )/2,l)$ where *h*, *k*, *l* are integers, as shown in Fig. 1(b). The parameter $\varepsilon$ is known as the incommensurability, with the average charge-stripe spacing given by $\mathrm{1/}\varepsilon$. As LSNO is tetragonal, including La_2_NiO_4.11_^13^, there is a second charge-stripe twin with stripes running at 90° within the Ni-O plane to those show in Fig. 1(a), producing the two magnetic Bragg reflections shown in Fig. 1(b). For charge-stripes with one hole per Ni site we should expect that the hole doping level determines $\varepsilon ={n}_{h}$, but it has been shown that $\varepsilon$ is systematically closer to 1/3 than *n*_*h*_^15,17^. The spins of the Ni^3+^, $S=1/2$ charge-stripe electrons themselves are known to have gapped quasi-one dimensional antiferromagetic excitations along the charge-stripes (q-1D) for energies below 10 meV with a dispersion that is doping independent below half doping^18–20^. For the $x=1/3$ the spins of q-1D excitations have been found to preferentially fluctuate in the out of Ni-O plane direction, with a $1.6\pm 0.1\,{\rm{meV}}$ energy width that corresponds to a 0.4 picosecond correlation time^18–20^. The gapped nature of the q-1D excitations contrasts with the gapless spin wave dispersion of the magnetically ordered Ni^2+^, $S=1$ in the spin stripes, that disperses up to 120 meV in La_2_NiO_4.11_^14,20^.

## Results

**Figure 2:**
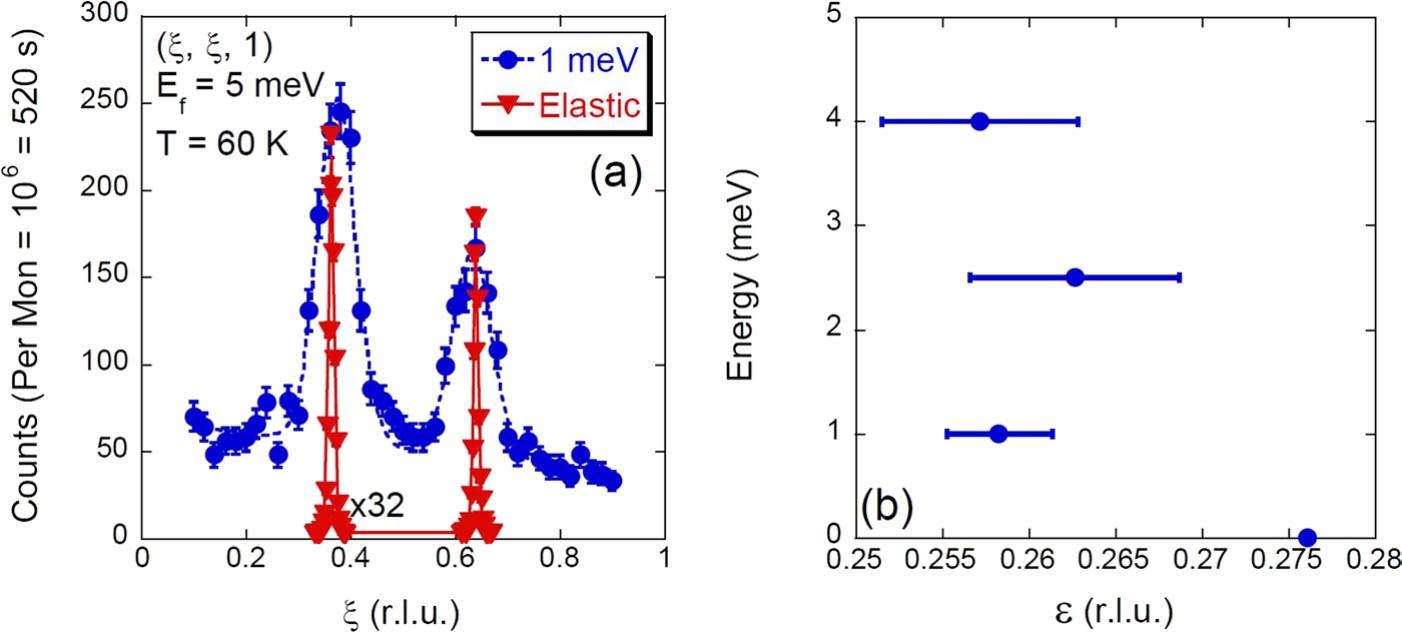
Magnetism of La_2_NiO_4.11_. (**a**) The magnetic Bragg reflections (elastic) and low energy magnetic excitations from the ordered Ni^2+^ $S=1$ spins (1 meV) of charge-stripe ordered La_2_NiO_4.11_. Dashed and solid lines are the results of fitting two Gaussians on sloping background to the two data sets. The scans indicate an offset in wave vector centring between the magnetic excitations and magnetic Bragg reflections, clearly both magnetic Bragg peaks are further away from $(0.5,0.5,1)$ than the centre of the magnetic excitations. The half width at half maximum resolution parallel to the scan direction was 0.0034 r.l.u. for the elastic scattering and 0.010 r.l.u. for the 1 meV scan. In (**b**) we show the incommensurability versus energy obtained from the magnetic Bragg reflections and magnetic excitations at the two positions shown in (**a**).

**Figure 3:**
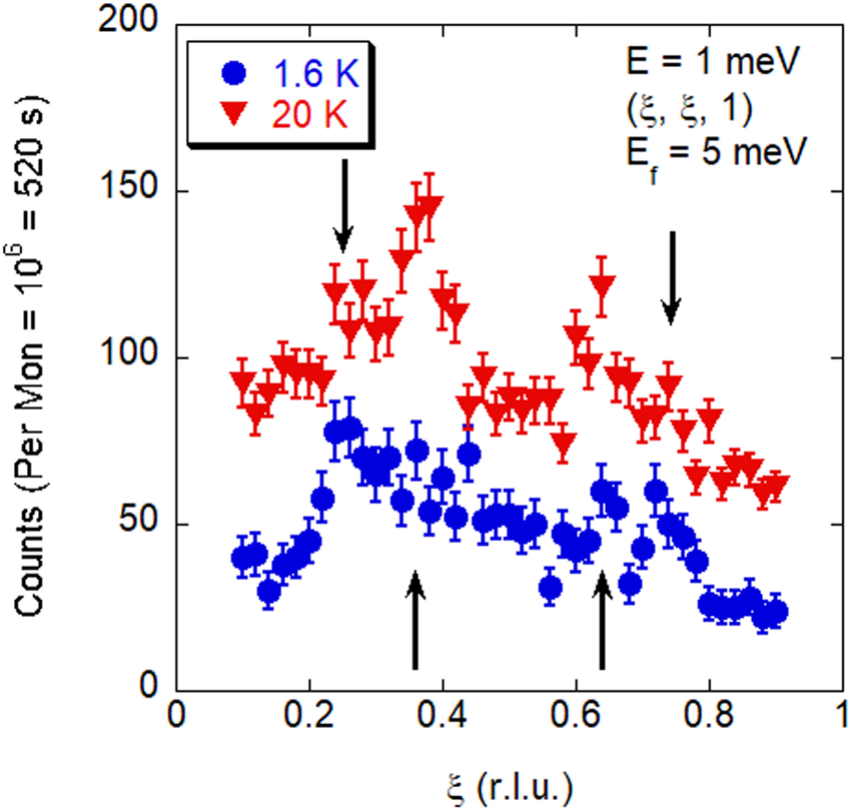
The magnetic excitations at 1 meV of charge-stripe ordered La_2_NiO_4.11_, at 1.6 K and 20 K. The 20 K data have been offset by the addition of 40 counts. The upward pointing arrows indicate the wave vector at which excitations from the ordered Ni^2+^, $S=1$ spins would be expected to occur as Goldstone modes of the spin stripe magnetic order, and the downward pointing arrows indicate the wave vector at which the q-1D excitations from the $S=1/2$ charge-stripe electrons occur. Between the two temperatures the excitations from the ordered moment gain intensity, whereas the excitations from the gapped q-1D excitations lose intensity as expected.

Neutron diffraction scans of the magnetic Bragg peak such as those shown in Fig. 2 were used to determine the spin ordering temperature of La_2_NiO_4.11±0.01_ to be ${T}_{nSO}=125\pm 5\,{\rm{K}}$. The charge-stripe ordering temperature is expected to be ${T}_{CO}\sim 150$, K^15^. The temperature dependence of the q-1D excitation has been established in LSNO for $x=1/3$, where the mode loses 90% of its intensity by 60 K^21^. A lower resolution study had previously established the presence of q-1D excitation in the same La_2_NiO_4.11±0.01_ crystal studied here^22^, and the dispersion of the q-1D excitation has been observed to be doping independent in charge-stripe ordered LSNO^19^. To ensure that we are accounting for the presence of the q-1D excitation in La_2_NiO_4.11±0.01_, we scanned by inelastic neutron scattering the temperature dependence of magnetic excitations along $(\xi ,\xi ,1)$ at 1 meV. In Fig. 3 we show how at 1.6 K we observe the ungapped magnetic excitations from the Ni^2+^ spins and the gapped q-1D excitations from the charge-stripe electrons. Consistent with our studies of the q-1D in LSNO $x=1/3$^18,21^, by 20 K the intensity of the gapped q-1D excitations appears to be reduced with no indication of a change in wave vector.

The temperature evolution of magnetic excitations is determined by non-linear terms in the quasiparticle Hamiltonian, involving processes such as magnon-magnon scattering, Umklapp processes, boundary scattering, etc.^23^. Reducing the dimensionality of a system increases the probability of magnon-magnon scattering as there is less phase-space for the magnons to disperse into. For gapped magnetic excitations once the temperature is above the energy of the spin gap the number of thermally activated magnons increases, increasing the probability of magnon-magnon scattering. Increased magnon-magnon scattering in turn increases the decay rate of magnons, causing a broadening of the magnetic excitations and reduction in their amplitude by neutron scattering. This effect has been established in quasi-one dimensional $S=1/2$ gapped quantum magnets, and spin Haldane chains to temperatures at least 1.5 times the energy of the zero temperature spin gap, with the spin gap increasing in size with increasing temperature^24^. The base temperature spin gap of the q-1D excitations was determined in LSNO $x=1/3$ to be $1.40\pm 0.07\,{\rm{meV}}={\rm{16}}\,{\rm{K}}$^20^, so at 20 K a loss in intensity of the q-1D excitation is as expected. The observed temperature dependence of the q-1D excitation in LSNO $x=1/3$ at *E* = 2 meV and 5 meV is however consistent with the dispersion of the q-1D being independent of temperature^18,21^. A comprehensive understanding of the temperature evolution of the gapped q-1D excitations in LSNO would first require accounting for the large energy width of the excitation at base temperature, and determmining what is the nature of the spin object that causes the 4 Ni-Ni periodicity along the charge-stripes of the gapped Q-1D excitations^18,19,21^.

In Fig. 2(a) we show at 60 K a scan of two magnetic Bragg peaks split from $(0.5,0.5,1)$ from a single stripe domain, where charge-stripes are orientated as shown in Fig. 1(a), and a similar scan of the magnetic excitations at 1 meV. In both cases the data were fitted by a least square fitting routine weighted to the experimental errors to a function of two Gaussian peaks on a sloping background. For triple axis spectromneters in focused or flat scattering geometry the Gaussian line shape has been shown to represent the resolution of the instrument^25^. At 1 meV the scan did not show any indication of the q-1D excitation, consistent with the small intensity of the q-1D observed at 60 K in LSNO $x=1/3$^21^. Any unaccounted residual intensity of the q-1D excitation would shift the fitted centre of the Gaussians away from the $(0.5,0.5,1)$ wave vector. The centres of the magnetic excitations are closer to $(0.5,0.5,1)$ than the magnetic Bragg peaks, similar to the offset observed in LCO+O^10^, see Table 1. The magnetic Bragg peaks are observed at the wave vectors $(\xi ,\xi ,1)$, $\xi =0.36330(6)$ and $\xi =0.63936(5)$ compared to the magnetic excitations at 1 meV that occur at $\xi =0.3743(18)$ and $\xi =0.6325(25)$. We can obtain $\varepsilon$ for both the magnetic order and the magnetic excitations from the splitting of the two centres, with $\varepsilon (0\,\,meV)=0.2761(1)$ corresponding to a real space periodicity of 19.8 Å and $\varepsilon (1\,\,meV)=0.258(3)$ corresponding a real space periodicity of 21.1 Å. In Fig. 2(b) we show how the $\varepsilon$ varies with energy transfer for the magnetic excitations compared to the magnetic Bragg reflections, obtained from the positions of the two centres shown in Fig. 2(a). Consistent with our earlier study of the spin wave dispersion of La_2_NiO_4.11±0.01_, we observe the magnetic excitations to be dispersionless at these low energies^14^.

**Table 1:** Fitted wave vector centres of the magnetic Bragg reflections, magnetic excitations at $E=1$ meV and $\varepsilon$ obtained from the wave vector difference of the two centres in scans along $(\xi ,\xi ,1)$ of charge-stripe ordered La_2_NiO_4.11_, from the fits shown in 2.

	(*ξ*, *ξ*, 1) (r.l.u.)		
	*ξ* Magnetic Bragg reflection	*ξ E* = 1 meV	Δ*ξ*
$(0.5-\varepsilon /2,0.5-\varepsilon /2,1)$	0.36330(6)	0.3743(18)	0.011(2)
$(0.5+\varepsilon /2,0.5+\varepsilon /2,1)$	0.63936(5)	0.6325(25)	0.0069(25)
$\varepsilon$	0.27606(8)	0.2582(31)	—

The error on the fitted centres is indicated by the digit(s) stated in the bracket.

The fractional difference in $\varepsilon$ we observe between the magnetic Bragg reflections and magnetic excitations in La_2_NiO_4.11_ is 6.5 ± 1.1%, which is close to the fractional differences of *δ*_*K*_ and *δ*_*h*_ of 7 ± 1% in LCO+O^10^. In the study of LCO+O the authors showed how this effect can not be produced by misalignment, and as La_2_NiO_4.11_ is tetragonal there is no structural twinning to take into account, unlike LCO+O^10^. After correcting the observed half width at half maximum (HWHM) of the elastic peak for instrument resolution, we used the inverse HWHM to determined the spin correlation length perpendicular to the charge-stripe direction of 135 ± 3.5 Å. The instrument resolution at 1 meV was calculated using the RESTRAX program using Cooper and Nathans formalism with Monte-Carlo ray-tracing simulations^26^. Using this resolution width we corrected the observed HWHM by quadrature, to obtain an intrinsic HWHM for the magnetic excitations at 1 meV. The inverse of this intrinsic HWMH gives a correlation length of 12 ± 1 Å perpendicular to the charge-stripe direction for the magnetic excitations at 1 meV.

**Figure 4:**
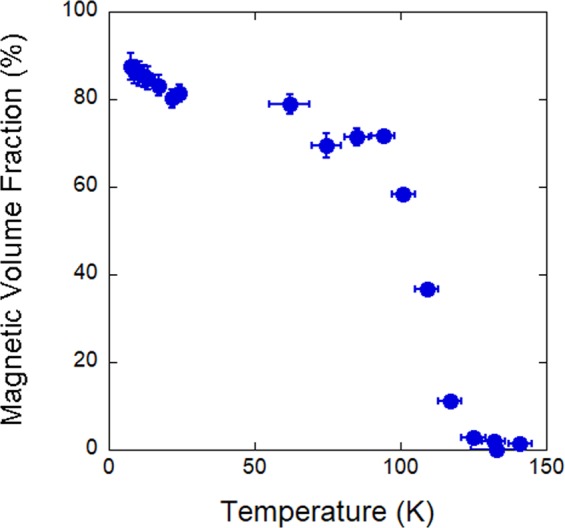
The magnetic volume fraction of La_2_NiO_4.11_. The magnetic volume fraction was determined from a weak transverse field *μ*SR measurement.

A weak transverse field *μ*SR measurement was performed to determine the magnetic volume fraction in an as grown La_2_NiO_4.11±0.01_ single crystal. Figure 4 shows the temperature dependence of the magnetic volume fraction. At 7 K, 87% of the sample is magnetically ordered, falling to 79% by 60 K. In tetragonal LSNO there are the two charge-stripe twins with the charge-stripes rotated by 90° between the two twins^14^. Consistent with a finite spin correlation length, and packing of two charge-stripe twins a magnetic volume fraction below 100% is observed. In our previous *μ*SR studies of charge-stripe ordered La_1.55_Sr_0.45_NiO_4_ we considered the asymmetry spectrum indicated such a high magnetic volume fraction that the material was fully magnetically ordered, yet the observed volume fraction is likely not to be below 100%^27^.

We measured the temperature dependence of the magnetic excitations, at 1 meV by cold neutron scattering on RITA-II, and at 4 meV, 6 meV by thermal neutron scattering on PUMA^28^. The intensity of magnetic excitations from a magnetically ordered phase should vary with temperature as:

1 $$I(\omega ,T)=I(0K)\times (n(\omega ,T)+1)\times {V}_{Mag}$$

where *V*_*Mag*_ is the magnetic volume fraction, and $n(\omega ,T)$ is the Bose factor:

2 $$n(\omega ,T)=\frac{1}{\exp (\hslash \omega /{k}_{B}T)-1}.$$

In Table 2 we show how the observed gain in integrated intensities of the magnetic excitations on warming to 60 K at 1 meV, 4 meV and 6 meV is accounted for by the Bose factor correction and the change in magnetic volume fraction. The temperature dependence of these magnetic excitations are therefore consistent with the magnetic excitations coming from the magnetically ordered volume of the material.

**Table 2:** The integrated intensity of the low energy magnetic excitations of La_2_NiO_4.11_, $E=1\,{\rm{meV}}$ taken on RITA-II with *k*_*f*_ = 1.5 Å^−1^, *E* = 4 meV and *E* = 6 meV taken on PUMA with *k*_*f*_ = 2.662 Å^−1^, note that the arbitrary units are experiment specific.

Energy	Cente (r.l.u.)	Integrated Intensity (arb. units)				
	(*h*, *h*1)	1.6 K	4.2 K	20 K	Observed 60 K	Corrected 60 K
1 meV	0.37	1.06(0.30)			7.2(0.6)	1.15(0.10)
1 meV	0.64	0.69(0.27)			4.4(0.5)	0.70(0.08)
4 meV	0.37		21.8(3.0)		46.2(2.3)	22.4(1.1)
4 meV	0.64		19.6(3.1)		43.9(2.6)	21.3(1.3)
6 meV	0.37			29.9(2.2)	48.6(3.2)	32.4(2.1)
6 meV	0.64			26.6(2.0)	40.8(3.1)	27.2(2.1)

Fitting of the base temperature data at 1 meV and 4 meV included a fit of the q-1D excitations, whereas the intensity of the q-1D excitations was assumed to be zero at 20 K for *E* = 6 meV. Integrated intensities at the lower temperature have been corrected for the Bose factor enhancement. The error of the integrated intensity is indicted in brackets. If the observed intensities are from excitations of the magnetically ordered volume, then they can be corrected for the reduction in the magnetic volume and the Bose factor to give the same intensity as at the lower temperature, as shown to be the case in the last column. Due to the low counting statistics in the scan of the magnetic excitations at 1 meV at 1.6 K, see Fig. 3, to achieve a meaningful fit the centre and peak width of the excitations from the ordered Ni^2+^, *S* = 1 spins were fixed to the fit values from 60 K, whereas the fit parameters of the q-1D excitations were free to vary.

If part of the magnetic excitation spectrum of La_2_NiO_4.11±0.01_ was coming from the magnetically ordered phase, and another part of the spectrum was coming from the far smaller magnetically unordered phase, there would be a discontinuity in the magnetic excitation spectrum. Previously we however observed the intensity and dispersion of the magnetic excitation spectrum of this single crystal of La_2_NiO_4.11±0.01_ varies smoothly and continuously with energy and wave vector^14^.

Considering these observations together we conclude that the full magnetic excitation spectrum comes from the magnetically ordered volume of La_2_NiO_4.11±0.01_.

## Discussion

Our observations indicate that in charge-stripe ordered La_2_NiO_4.11±0.01_, the lowest energy magnetic excitations are from the magnetically ordered volume of the material. We therefore must conclude that unlike what is observed in LCO+O by Jacobsen and coworkers, the wave vector difference between the low energy magnetic excitations and magnetic Bragg reflections specifically in La_2_NiO_4.11±0.01_ has a different origin than ‘electronic phase separation’^10^.

An offset in wave vector of the magnetic Brag reflections and magnetic excitations on going to zero energy transfer means that the magnetic excitations cannot simply be regarded as Goldstone modes of the magnetic order. The success of linear spin wave theory in modelling the magnetic excitations of LSNO as though they are Goldstone modes however, suggests that we are missing a perturbative term(s) in our description of the magnetism of La_2_NiO_4.11±0.01_^4,14^.

Linear spin wave theory is used to calculate the magnetic excitations from a long range magnetic order, yet LSNO has short ranged magnetic order^15^. It may therefore be important to consider a relaxation of the wave vector of the magnetic excitations that propagate into the unordered magnetic volume. This could cause a shift of the average wave vector centring of the magnetic excitations. This proposition can be investigated by studying doping levels with distinctly different magnetic correlation lengths^15^.

Alternatively our model of the spin excitation spectrum may not include all the spin interactions, missing a weak perturbative spin interaction. It has been noted in the parent cuprate material La_2_CuO_4_ there is a weak Dzyaloshinskii Moriya interaction which introduces an antisymmetric spin exchange interaction, caused by the buckling of the Cu-O-Cu bonds^29^. The crystal structure of La_2_NiO_4.11±0.01_ is high temperature tetragonal, a phase in which there is no collective tilting of Ni-O octahedra, unlike the ordered tilting arrangement of the octahedra in the low temperature tetragonal or low temperature orthorhombic phases of La_2_NiO_4_^13,30^. A coherent effect from Dzyaloshinskii Moriya interaction in La_2_NiO_4.11±0.01_ therefore appears an unlikely cause of the wave vector difference we observe here, as there is no coherent buckling of the Ni-O-Ni bonds in the high temperature tetragonal phase. In magnetically ordered materials with many spin interactions, that have gapped spin wave spectra, it has been observed that the minimum of their spin waves can be well separated from the wave vector of their magnetic Bragg peaks. Examples of this are found in incommensurately magnetically ordered BaCo_2_(AsO_4_)_2_, or the commensurately ordered LiNiPO_4_ a material that is close to a spiral ordered magnetic state^31,32^. We note that a recent theoretical study predicts a vector chiral order in La_2−*x*_Sr_*x*_CuO_4_ at low doping, this theory is based on the holes forming vortex and antivortex polaron pairs that order as topological dipoles into electronic polymer phases^33^. As this theoretical approach produces a vector chiral order, this approach offers a promising avenue of research to understand the offset we see in this study and the periodicity of the gapped q-1D magnetic excitations in LSNO. Present charge-stripe ordered modelling of the spin wave excitations spectrum of LSNO indicate that additional spin interactions are too weak to accurately determine from published studies, but there may be additional weak spin interactions in LSNO that are yet to be accounted for, a potential a cause of the observed difference in wave vector^4,14^.

## Conclusions

There is a wave vector difference between the centring of magnetic Bragg reflections and lowest energy magnetic excitations in charge-stripe ordered La_2_NiO_4.11±0.01_. We have shown how the magnetic excitations occur in the magnetically ordered volume of the material, with 87% of the material being magnetically ordered at base temperature. Further experimental and theoretical studies are required to elucidate the origin of the wave vector difference between the magnetic Bragg reflections and the lowest energy magnetic excitations in La_2−*x*_Sr*x*NiO_4+*δ*_ observed in this study. Whether the cause of the wave vector offset in La_2−*x*_Sr*x*NiO_4+*δ*_ is the same as in LCO+O, remains an open question^10^.

## Methods

We studied a single crystal of La_2_NiO_4.11±0.01_^34^, on the triple axis spectrometers RITA-II^20^ and PUMA^28^. This is the same crystal that we have previously used to study the spin wave dispersion up to 120 meV^14^. The oxygen content of an as grown crystal has been determined destructively by thermo-gravimetric analysis^34^. In comparison with published literature, the charge-stripe order structure of our La_2_NiO_4.11±0.01_ sample is consistent with having 0.11 < *δ* < 0.125^16,35^. The data were collected with a fixed final neutron wave vector of *k*_*f*_ = 1.5 Å^−1^ on RITA-II and of *k*_*f*_ = 2.662 Å^−1^ on PUMA. On RITA-II a beryllium filter, and on PUMA a pyrolytic graphite (PG), was placed between the sample and analyzer to suppress higher-order harmonic scattering. On both instruments the neutrons final and initial energy was selected by Bragg reflection from a pyrolytic graphite (PG) monochromator (vertically focused on RITA-II and double focusing on PUMA) and a PG analyzer (doubly focused on PUMA, flat for diffraction and horizontally focused for excitations on RITA-II). The sample was mounted in a standard orange cryostat on RITA-II, and a closed cycle refrigerator on PUMA. On both instruments the sample was orientated so that $(h,h,l)$ positions in reciprocal space could be accessed, and we reference reciprocal space with the tetragonal unit cell parameters *a* = *b* = 3.86 Å, *c* = 12.6 Å for the I4/mmm space group. The scattering geometry used on both instruments was the w-configuration, with scattering at sample position being in the opposite angular direction to that at the analyser and monochromator.

Weak transverse field *μ*SR measurements were performed on the HiFi spectrometer at the ISIS facility, to determine the magnetic volume fraction in as grown La_2_NiO_4.11±0.01_ single crystal. The crystal was crushed and mounted on the HiFi CCR on a silver backing plate. The measurement determined the initial asymmetry of La_2_NiO_4.11±0.01_ on warming. Missing initial asymmetry observed at a pulsed muon source in a magnetic sample is from the fraction of muons which stop in a magnetic environment that depolarizes the muons. This signal was converted into the magnetic volume fraction after performing standard corrections for the experimental background.

## Data Availability

The data presented in this paper is available here: 10.17030/uclan.data.00000187.
